# Supplementary material for: The neural correlates of context driven changes in the emotional response: An fMRI study
Source: PLoS One. 2022 Dec 30;17(12):e0279823. doi: 10.1371/journal.pone.0279823 (PMC9803168; doi:10.1371/journal.pone.0279823)
Supplement: S1 Fig — Coordinates are in Montreal Neurological Institute (MNI) space. Statistical maps were visualized on the MNI 152 template brain provided in MRIcroGL [39]. (DOCX) [file pone.0279823.s001.docx]

**Supporting information**

**The neural correlates of context driven changes in the emotional response: an fMRI study**


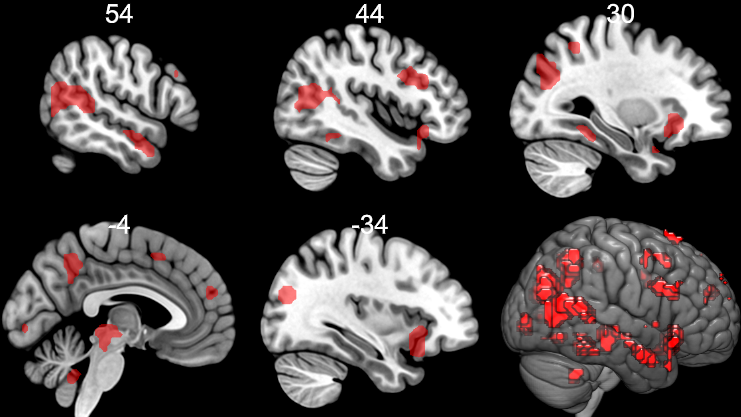


**S1 Fig.** **General context effect: full pictures with the context vs. firstly presented images (pictures without the context) at** **p< .05, family-wise error (FWE) corrected for multiple comparison.**

Coordinates are in the Montreal Neurological Institute (MNI) space. Statistical maps were visualized on the MNI 152 template brain provided in MRIcroGL [39].
