# Supplementary material for: The neural correlates of context driven changes in the emotional response: An fMRI study
Source: PLoS One. 2022 Dec 30;17(12):e0279823. doi: 10.1371/journal.pone.0279823 (PMC9803168; doi:10.1371/journal.pone.0279823)
Supplement: S1 Table — P1: First picture of the picture pairs is positive. N1: First picture of the picture pairs is negative. P2: Second picture of the picture pairs is positive. N2: Second picture of the picture pairs is negative. + Results of the bootstrapped paired t-test. ++ Results of the Wilcoxon Signed Rank Test. *p < .001. Valence and arousal were measured from 1 to 7 (1 being very unpleasant and 7 very pleasant; 1 being calm and 7 very excited, respectively). (DOCX) [file pone.0279823.s002.docx]

**Supporting information**

**The neural correlates of context driven changes in the emotional response: an fMRI study**

**S1 Table.** Valence and arousal values for the pictures of the post-task after scan by contrasts.

| Condition | Short name | First picture | Valence  Mean (SD) | Arousal  Mean (SD) | Second picture | Valence  Mean (SD) | Arousal  Mean (SD) | Changes in mean [95% CI]^+^  Comparison of the picture pairs by valence ^++^ | Changes in mean [95% CI]^+^  Comparison of the picture pairs by arousal^++^ |
| --- | --- | --- | --- | --- | --- | --- | --- | --- | --- |
| Shift | P1N2 | Positive  (P1) | 4.968  (0.787) | 3.097  (1.192) | Negative  (N2) | 2.306  (0.706) | 4.952  (1.133) | -2.66 [-3.01- -2.31]*  Z=4.785* | 1.85 [1.52-2.20] *  Z=4.863 * |
| Upregula-tion (Non-Shift) | P1P2 | Positive  (P1) | 5.850  (0.745) | 4.398  (1.137) | Positive  (P2) | 6.129  (0.716) | 4.903  (1.012) | 0.28 [0.18-0.39] *  Z=4.039* | 0.51 [0.30-0.71] *  Z=3.885* |
| Shift | N1P2 | Negative  (N1) | 3.575  (0.807) | 3.930  (1.178) | Positive  (P2) | 5.935  (0.463) | 4.925  (1.161) | 2.36 [2.06 -2.66]*  Z=4.866* | 0.99 [0.77-1.21]*  Z=4.789* |
| (Upregula-tion) Non-Shift | N1N2 | Negative  (N1) | 2.398  (0.498) | 4.307  (1.090) | Negative  (N2) | 1.882  (0.470) | 5.064  (0.886) | -0.52 [-0.65- -0.38]*  Z=-4.207* | 0.75 [0.56-0.97]  Z=-4.514* |

Note. P1: First picture of the picture pairs is positive. N1: First picture of the picture pairs is negative. P2: Second picture of the picture pairs is positive. N2: Second picture of the picture pairs is negative. ^+^ Results of the bootstrapped paired t-test. ^++^ Results of the Wilcoxon Signed Rank Test. *p<.001. Valence and arousal were measured from 1 to 7 (1 being very unpleasant and 7 very pleasant; 1 being calm and 7 very excited, respectively).
