# Supplementary material for: The neural correlates of context driven changes in the emotional response: An fMRI study
Source: PLoS One. 2022 Dec 30;17(12):e0279823. doi: 10.1371/journal.pone.0279823 (PMC9803168; doi:10.1371/journal.pone.0279823)
Supplement: S2 Table — P1: First picture of the picture pairs is positive. N1: First picture of the picture pairs is negative. P2: Second picture of the picture pairs is positive. N2: Second picture of the picture pairs is negative. Different letters (a, b, c) represent significant (p < .05) difference between mean scores, whereas the same letters represent non-significant difference between mean scores according to the paired post hoc test of repeated measure of ANOVA. (DOCX) [file pone.0279823.s003.docx]

**Supporting information**

**The neural correlates of context driven changes in the emotional response: an fMRI study**

**S2 Table.** Mean reaction times (in milliseconds) to the second picture in the scanner by the type of picture pairs in the Emotional Shifting Task.

| Picture pairs | Mean (SD) | Minimum | Maximum |
| --- | --- | --- | --- |
| P1N2_a_ | 1067.885 (347.974) | 528.67 | 2018.33 |
| P1P2_b_ | 789.732 (241.741) | 424.50 | 1357.50 |
| N1P2_b_ | 809.097 (263.533) | 407.67 | 1496.80 |
| N1N2_c_ | 977.138 (271.741) | 471.50 | 2294.40 |

*Note.* P1: First picture of the picture pairs is positive. N1: First picture of the picture pairs is negative. P2: Second picture of the picture pairs is positive. N2: Second picture of the picture pairs is negative. Different letters (a, b, c) represent significant (p < .05) difference between mean scores, whereas the same letters represent non-significant difference between mean scores according to the paired post hoc test of repeated measure of ANOVA.
