# Supplementary material for: The neural correlates of context driven changes in the emotional response: An fMRI study
Source: PLoS One. 2022 Dec 30;17(12):e0279823. doi: 10.1371/journal.pone.0279823 (PMC9803168; doi:10.1371/journal.pone.0279823)
Supplement: S3 Table — L = left; the initial statistical threshold was set to p< .05, family-wise error (FWE) corrected for multiple comparison. (DOCX) [file pone.0279823.s004.docx]

**Supporting information**

**The neural correlates of context driven changes in the emotional response: an fMRI study**

**S3 Table. General context effect:** Increased activations to the 2^nd^ pictures with context compared to the 1^st^ pictures without context

| Cluster size (voxel) | Region | Side | Peak T-values | MNI coordinates | | |
| --- | --- | --- | --- | --- | --- | --- |
|  |  |  |  | x | y | z |
| 167 | Raphe (brainstem) | L | 12.94 | -6 | -28 | -10 |
|  | Red nucleus (brainstem) | R | 6.78 | 6 | -19 | -7 |
| 201 | Precuneus | R | 11.61 | 0 | -55 | 44 |
| 39 | Calcarine | L | 10.68 | -9 | -88 | -1 |
| 387 | Middle Temporal Gyrus | R | 9.76 | 48 | -61 | 11 |
|  | Superior Temporal Gyrus | R | 9.63 | 57 | -46 | 17 |
| 105 | Middle Occipital Gyrus | L | 9.47 | -30 | -79 | 23 |
|  | Superior Occipital Gyrus | L | 8.67 | -24 | -70 | 26 |
|  | Superior Occipital Gyrus | L | 6.80 | -21 | -70 | 38 |
| 145 | Middle Occipital Gyrus | R | 9.45 | 30 | -73 | 32 |
| 244 | Middle Temporal Gyrus | L | 9.37 | -57 | -58 | 17 |
|  | Middle Occipital Gyrus | L | 8.62 | -45 | -76 | 5 |
|  | Middle Temporal Gyrus | L | 8.34 | -51 | -67 | 14 |
| 84 | Middle Temporal Gyrus | R | 9.29 | 60 | 2 | -22 |
| 29 | Inferior Parietal Gyrus | R | 9.06 | 33 | -52 | 53 |
| 145 | Insula | L | 8.80 | -33 | 20 | -7 |
|  | Posterior Orbital Gyrus | L | 7.81 | -36 | 20 | -19 |
|  | Insula | L | 6.96 | -27 | 11 | -19 |
| 120 | Inferior Frontal Gyrus, opercular part | R | 8.44 | 42 | 14 | 26 |
|  | Inferior Frontal Gyrus, triangular part | R | 7.77 | 45 | 23 | 26 |
| 37 | Inferior Temporal gyrus | L | 8.42 | -42 | -43 | -19 |
| 27 | Precentral Gyrus | L | 8.40 | -39 | 2 | 38 |
| 83 | Fusiform Gyrus | R | 8.13 | 42 | -43 | -19 |
|  | Fusiform Gyrus | R | 7.74 | 33 | -40 | -16 |
| 142 | Inferior Frontal Gyrus, orbital part | R | 7.97 | 33 | 23 | -7 |
|  | Posterior Orbital Gyrus | R | 7.46 | 42 | 23 | -19 |
|  | Superior Temporal pole | R | 7.01 | 39 | 20 | -31 |
| 24 | Calcarine | R | 7.97 | 15 | -91 | 2 |
| 28 | Lingual gyrus | L | 7.83 | -24 | -64 | -10 |
| 41 | Vermis |  | 7.52 | 0 | -52 | -37 |
| 54 | Supplementary Motor Area | R | 7.22 | 6 | 11 | 53 |
|  | Supplementary Motor Area | L | 6.74 | -6 | 11 | 53 |
| 21 | Supplementary Motor Area | R | 7.13 | 6 | 23 | 65 |
| 35 | Superior Frontal Gyrus, medial | L | 6.95 | -6 | 53 | 23 |
|  | Pregenual Anterior Cingulate Cortex | R | 6.36 | 6 | 47 | 17 |
| 32 | Inferior Parietal Gyrus | L | 6.89 | -27 | -55 | 47 |
| 17 | Middle Frontal Gyrus | R | 6.64 | 36 | 2 | 53 |
| 17 | Postcentral Gyrus | L | 6.62 | -42 | -34 | 47 |
| 10 | Superior Frontal Gyrus | R | 6.46 | 15 | 59 | 35 |
|  | Superior Frontal Gyrus, medial | R | 5.87 | 6 | 59 | 38 |
| 10 | Lingual Gyrus | L | 6.29 | -24 | -46 | -7 |

Note. L=left; the initial statistical threshold was set to p< .05, family-wise error (FWE) corrected for multiple comparison
